# Toegankelijk monitoronderzoek voor mensen met een lichte verstandelijke beperking en/of laaggeletterden

**DOI:** 10.1007/s12508-024-00430-4

**Published:** 2024-03-12

**Authors:** Monique C. J. Koks-Leensen, Anouk Menko, Fieke Raaijmakers, Kirsten E. Bevelander

**Affiliations:** Academische werkplaats Sterker op eigen benen, afdeling Eerstelijnsgeneeskunde, Radboudumc, Nijmegen, Nederland; Academische werkplaats AMPHI Integraal, Gezondheidsbeleid, afdeling Eerstelijnsgeneeskunde, Radboudumc, Nijmegen, Nederland; Academische werkplaats AMPHI Integraal, Gezondheidsbeleid, afdeling Eerstelijnsgeneeskunde, Radboudumc, Nijmegen, Nederland; Veiligheids- en Gezondheidsregio Gelderland Midden, Arnhem, Nederland; Academische werkplaats Sterker op eigen benen, afdeling Eerstelijnsgeneeskunde, Radboudumc, Nijmegen, Nederland; Academische werkplaats AMPHI Integraal, Gezondheidsbeleid, afdeling Eerstelijnsgeneeskunde, Radboudumc, Nijmegen, Nederland

**Keywords:** inclusief onderzoek, cocreatie, lichte verstandelijke beperking, laaggeletterdheid, monitoronderzoek, gezondheidsverschillen

## Abstract

Mensen met een lichte verstandelijke beperking (LVB) en laaggeletterden hebben een verhoogd risico op gezondheidsproblemen. Zij worden echter onvoldoende bereikt met gezondheidsonderzoek, waardoor inzicht in hun gezondheid en ondersteuningsbehoeften ontbreekt. Tijdens de COVID-19-pandemie werd de essentie van dit inzicht voor passend beleid extra duidelijk. Wij hebben een inclusief monitoronderzoek uitgevoerd met een aangepaste vragenlijst om de impact van de COVID-19-pandemie op de gezondheid van mensen met LVB en/of laaggeletterden te monitoren. In dit artikel beschrijven we onze inclusieve aanpak en delen we succesfactoren en tips voor toekomstig gezondheidsonderzoek.

## Inleiding

Gezondheidsonderzoek, waaronder onderzoek gericht op de publieke gezondheid, bereikt niet iedereen. Mensen met een lichte verstandelijke beperking (LVB) en mensen die laaggeletterd zijn, zijn structureel ondervertegenwoordigd [1]. Vaak zijn de (wervings)methoden te ingewikkeld en te veel ingesteld op (geschreven) taal, waardoor te weinig zicht is op hun gezondheid en ondersteuningsbehoeften. Dit is opmerkelijk, want hun slechtere gezondheid in vergelijking met de reguliere bevolking geeft al lange tijd reden tot zorg [2].

Tijdens de COVID-19-pandemie werd het belang van dergelijk gezondheidsonderzoek snel duidelijk, aangezien specifieke kennis over deze doelgroep ontbrak en daarom niet bij het vormgeven van beleid meegenomen kon worden. Een belangrijke reden hiervoor was dat deze subgroepen onvoldoende werden bereikt met het landelijke onderzoek van de RIVM Coronagedragsunit naar de impact van COVID-19 [3]. Om inzicht te krijgen in de impact die de COVID-19-pandemie op deze groepen in kwetsbare posities heeft, ontwikkelden we met en voor mensen met LVB en/of laaggeletterden daarom een aangepaste monitor.

Met deze aangepaste monitor bereikten we een groep deelnemers die significant verschilde van een steekproef uit de reguliere GGD-monitor die dezelfde aangepaste monitor invulde. Eerstgenoemden waren significant lager opgeleid, hadden vaker een migratieachtergrond, vaker ondersteuning in hun dagelijks leven en minder vaak betaald werk, en gingen vaker naar de dagbesteding. Mensen met LVB en/of laaggeletterden ervaarden een grotere impact van de COVID-19-pandemie dan mensen uit de reguliere steekproef [4]. Ze rapporteerden vaker negatieve gevoelens tijdens de pandemie, hadden meer moeite om de informatie over COVID-19 te begrijpen en om gezondheidsgedrag ter preventie van infectieziekteverspreiding toe te passen.

De aangepaste monitor werd gedurende de pandemie drie keer afgenomen (*n*= 412 eerste ronde, *n*= 351 tweede ronde en *n*= 296 derde ronde), volgens een herhaald crosssectioneel onderzoeksontwerp. De surveyrondes vonden plaats tussen november 2020 en november 2021, op meetmomenten waarop de maatregelen verder versoepeld waren. Hieruit bleek dat de mentale gezondheid van beide deelnemersgroepen verbeterde, al bleef die van mensen in kwetsbare posities achter bij de reguliere steekproef [4]. Met de aangepaste monitor maakten we dus een nieuw en aanvullend perspectief inzichtelijk voor beleidsmakers en praktijkprofessionals.

In deze bijdrage geven we een korte procesbeschrijving van de ontwikkeling en implementatie van deze aangepaste monitor. Ook staan we stil bij de succesfactoren die relevant zijn voor toekomstig gezond-heidsonderzoek. We hopen hiermee anderen te inspireren om alternatieve ontwerp-, wervings- en afname-methoden toe te passen gericht op mensen die doorgaans niet met gezondheidsonderzoek bereikt worden, om zo een meer complete en representatieve onderzoekspopulatie te verkrijgen.

## Inclusieve ontwikkeling van de aangepaste monitor

### Multidisciplinair en inclusief team

Voor dit traject vormde inclusief onderzoek het uitgangspunt: onderzoek waarbij mensen om wie het gaat in alle onderzoeksfasen actief betrokken worden [5]. Gedurende het hele project hebben we intensief samengewerkt met co-onderzoekers en ervaringsdeskundigen met LVB of laaggeletterdheid [6, 7], én met verschillende praktijkprofessionals, zoals beleidsmakers en gezondheidsprofessionals van de GGD’en Gelderland-Midden en -Zuid, MEE Gelderse Poort, Pharos en diverse zorgorganisaties.

Ervaringsdeskundigen zijn mensen met LVB en/of laaggeletterden die opgeleid zijn om hun ervaringen te delen met anderen en te spreken namens de groep die zij vertegenwoordigen. Zij hebben op verschillende momenten advies gegeven. Co-onderzoekers zijn ook ervaringsdeskundigen, maar met specifieke onderzoekstaken en -vaardigheden, die gedurende het hele onderzoek deel uitmaken van het onderzoeksteam.

### Ontwikkeling inclusieve monitor

Om tijdens perioden met strengere sociale restricties toch een zo groot mogelijke groep deelnemers te bereiken, kozen we een online toepassing. We maakten gebruik van de bestaande infrastructuur van het toegankelijke online platform www.ikonderzoekmee. nl voor burgerwetenschap, specifiek ontwikkeld voor mensen met LVB en/of laaggeletterden. Dit platform beschikt over een vragenlijsttool met duidelijke ant-woordschalen, een rustige lay-out en ondersteunende technologie, zoals een tekst-naar-spraakfunctie. Met verkregen feedback tijdens het project werd het platform doorontwikkeld. Zo hebben we een spraaknaar-tekstfunctie toegevoegd om het beantwoorden van open vragen te faciliteren, naast de mogelijkheid om gifjes in te voegen die deelnemers stimuleren door te gaan met de vragenlijst.

De basis voor de eerste vragenlijst vormde de landelijke survey van de RIVM Coronagedragsunit naar de impact van COVID-19 [3]. In cocreatie met de ervaringsdeskundigen werd in drie stappen een toegankelijke en begrijpelijke versie voor mensen met LVB en/of laaggeletterden ontwikkeld [8]: 1) de survey werd ingekort, 2) het aantal antwoordcategorieën werd verminderd en 3) het taalniveau werd aangepast. In opeenvolgende surveyrondes werd deze vragenlijst verder aangepast, steeds volgens dezelfde ontwikkelingsstappen. Een projectgroep van praktijkprofessionals en co-onderzoekers dacht mee over relevante onderwerpen en selecteerde vragen die pasten bij het projectdoel en de doelgroep. Daarna werden samen met de co-onderzoekers de antwoordcategorieën gecontroleerd en het taal- en abstractieniveau van de vragen aangepast.

De online conceptvragenlijst werd getest in cognitieve interviews met steeds vier tot zes ervaringsdeskundigen. In deze interviews worden deelnemers uitgenodigd hardop na te denken bij het beantwoorden van de vragen, om zo te ontdekken of ze de vragen en antwoordschalen interpreteren zoals bedoeld is. Op basis van deze interviews werden laatste aanpassingen aangebracht in de vragen en de antwoordschalen. Ten slotte deden twee andere ervaringsdeskundigen een gebruikerstest, waarbij ze werden geobserveerd tijdens het invullen van de online survey. De uiteindelijke ‘makkelijk lezen’-vragenlijst bestond steeds uit veertig tot zestig vragen, afhankelijk van de routing.

### Wervingsstrategie en afname van de survey

Om potentiële deelnemers te bereiken werden de surveys verspreid via zogenaamde ‘vindplaatsen’ van mensen met LVB en/of laaggeletterden. Dit waren organisaties die met deze doelgroep(en) werken, zoals belangenorganisaties, zorginstellingen voor mensen met LVB, ROC’s, bibliotheken, sociale werkplaatsen, enzovoort. Daarvoor werd ‘makkelijk lezen’-wervingsmateriaal ontwikkeld en gebruikt, zoals flyers, aangepaste advertenties en informatievideo’s.

De online vragenlijst stond steeds vier tot zes weken open, waardoor organisaties de tijd kregen om deze binnen hun netwerk te verspreiden. Het invullen van de monitor gebeurde anoniem. Voor mensen die niet in staat waren om de survey zelfstandig in te vullen was persoonlijke ondersteuning beschikbaar. Deze werd geboden door studenten social work of pedagogiek en ervaringsdeskundigen. Voorafgaand aan de vragenlijstronde werden deze studenten door MEE Gelderse poort getraind om laaggeletterde deelnemers en/of deelnemers met LVB adequaat te kunnen ondersteunen bij het invullen van de vragenlijst. Over alle rondes heen heeft 47% van de deelnemers de monitor met ondersteuning ingevuld. Aan het einde van iedere survey werden deel-nemers gevraagd zich via een aparte online procedure aan te melden voor volgende surveyrondes. Dit resulteerde in een deel-nemerspanel dat in een volgende ronde rechtstreeks benaderd kon worden.

### Duiden en delen van resultaten

Na iedere ronde bespraken we de resultaten in vijf tot zes duidingssessies met praktijkprofessionals, beleidsmakers en ervaringsdeskundigen. In deze sessies reflecteerden we gezamenlijk op de resultaten om ze om te zetten in praktijkgerichte oplossingen en handelingsperspectieven. Deze konden we reeds tijdens het lopende project terugkoppelen aan alle betrokken stakeholders. Om de opbrengst breed te delen gaven we verscheidene presentaties en ontwikkelden we in cocreatie steeds twee factsheets – een voor professionals en een voor de doelgroep.

## Lessen voor de toekomst

### Succesfactoren uit het project

Dit project heeft mensen met LVB en/of laaggeletterden laten participeren in monitoronderzoek. Door iteratief te werken en regelmatig met betrokken experts en ervaringsdeskundigen te evalueren, kwam in dit project een aantal succesfactoren naar voren dat belangrijk is voor toekomstig onderzoek.

#### 1 Een inclusieve werkwijze loont

Met de gekozen aanpak zijn we erin geslaagd om mensen die niet regulier aan onderzoek kunnen meedoen wel te bereiken, zelfs tijdens periodes van lockdowns en sociale restricties. Deelnemers gaven aan dat ze op deze manier in staat gesteld werden om hun stem te laten horen, iets wat ze niet alleen als heel prettig, maar ook als noodzakelijk ervaarden. Het inclusieve onderzoek hebben we in dit project op verschillende manieren vormgegeven:

- betrokkenheid van een co-onderzoeker met LVB en een co-onderzoeker laaggeletterdheid (taalambassadeur) gedurende het gehele onderzoek; zij dachten mee over de inhoud van de vragenlijst en het werven van deelnemers, en werkten actief mee aan de vragenlijstontwikkeling en de duiding en de verspreiding van de resultaten. Op deze manier was het perspectief van de doelgroep steeds vertegen-woordigd in het project, waardoor de onderwerpen en de aanpak goed bij hen aansloten;
- samenwerking met ervaringsdeskundigen vanuit MEE, de STERKplaats LFB Nijmegen en stichting ABC: zij werkten mee aan het testen van vragenlijsten, het werven en ondersteunen van deelnemers en het duiden en verspreiden van de resultaten. Door meerdere mensen erbij te betrekken werd onze aanpak gekenmerkt door een breed doelgroepperspectief. Hierdoor verkregen we meer kennis over de doelgroepen en goede ingangen in hun netwerk, en groeide dankzij de passende monitor en aanpak hun vertrouwen;
- samenwerken met praktijkprofessionals die veel met de doelgroep werken: in de multidisciplinaire projectgroep leverden deze experts input op onder andere de inhoud van de vragenlijst en het werven van deelnemers, waardoor we met de vragenlijsten ook goed aansloten bij praktijkbehoeften. Daar-naast werd een bredere groep professionals betrokken bij de duidingssessies. Door onze resultaten tussentijds met hen te delen, ontstond een goede verbinding tussen het onderzoek, het beleid en de praktijk, en konden ze de handelingsperspectieven tijdens het project al in praktijk brengen.

#### 2 Een passende wervingsmethode is cruciaal

Om mensen met LVB en/of laaggeletterden deel te laten nemen aan monitoronderzoek doen is meer nodig dan alleen een ‘makkelijk lezen’-vragenlijst – potentiële deelnemers moeten immers eerst bereikt worden op een manier die bij hen past. In dit project is daar op ingezet met werving via-via en gebruik van meerdere manieren van communicatie over het onderzoek. Mensen met LVB en/of laaggeletterden weten niet altijd wat onderzoek behelst, en de afstand tussen hen en de onbekende onderzoeker is groot. Daarom loont het om deelnemers zo veel mogelijk op een voor hen vertrouwde plek en via bekenden te werven. We hebben ons onderzoek zo breed mogelijk uitgezet via praktijkorganisaties en mensen uit de doelgroep zelf. Zij hadden als intermediairs tussen onderzoeker(s) en potentiële deelnemers een waardevolle rol.

We hebben daartoe met vindplaatsen – organisaties waar mensen uit onze doelgroep veel komen, zoals zorgorganisaties voor LVB, sociale werkplaatsen, ROC’s, taalhuizen en bibliotheken – samengewerkt en verschillende ervaringsdeskundigen gevraagd om de vragenlijst in hun netwerk te verspreiden. Dit vroeg echter wel wat van deze intermediairs; zij leverden inspanning om de beoogde doelgroep te bereiken, zonder direct voordeel voor henzelf. Vooral in de praktijk-organisaties waarin er tijdens de aanhoudende pandemie qua werkdruk al veel van deze mensen gevraagd werd, vroeg dit om extra tijdsinvestering.

Daarnaast gebruikten we verschillende wervings-kanalen en -materialen, ontwikkeld in samenwerking met experts en co-onderzoekers. Zo konden we diverse deelnemersgroepen passend aanspreken, onder andere via wervingsflyers, nieuwsberichten in magazines of op websites voor de doelgroepen, WhatsApp-berichten, en telefonisch of via mailing in geval van eerdere deelnemers. Naast deze ‘geschreven’ materialen ontwikkelden we op aanraden van betrokken ervaringsdeskundigen verschillende korte uitlegvideo’s, waarin (co-)onderzoekers meer uitleg geven over het project en het belang van deelname aan onderzoek.

#### 3 Een inclusieve monitor bereikt een bredere populatie

De inclusieve monitor is niet alleen bij mensen met LVB en/of laaggeletterden afgenomen, maar ook bij een steekproef van reguliere onderzoeksdeelnemers van twee GGD’en. De reguliere deelnemers werden in de laatste vragenlijstronde gevraagd naar hun mening over de ‘makkelijk lezen’-vragenlijst. Een groot deel (78% van de respondenten die deze vraag beantwoordden) vond het prima als ze vaker een derge-lijke vragenlijst voorgelegd zouden krijgen, als daarmee laaggeletterden kunnen meedoen en een groter bereik gegenereerd wordt. Dat biedt mogelijkheden om een ‘makkelijk lezen’-vragenlijst breder in te zetten dan alleen voor mensen met LVB en/of laaggeletterden. Deze bevinding is relevant in het licht van maatschappelijke ontwikkelingen, zoals de afnemende leesvaardigheid in de Nederlandse samenleving, die het belang van het creëren van inclusief (gezondheids)onderzoek nog eens onderstrepen.

### Praktische tips

Op basis van de hierboven beschreven succesfactoren sluiten we af met een aantal praktische tips voor inclusief gezondheidsonderzoek in de praktijk.

#### 1 Maak het onderzoek inclusief

- Werk samen met zowel ervaringsdeskundigen als praktijkprofessionals om zo verschillende perspectieven in het onderzoek te betrekken en benutten. Dit verrijkt het onderzoek en maakt het makkelijker om aan te sluiten bij de behoeften van alle betrokkenen.
- Sluit zo veel mogelijk aan bij de belevingswereld van mensen die je wilt bereiken en bij je onderzoek wilt betrekken. Dat betekent deelnemers werven op plekken waar zij veel komen. En gebruikmaken van passende communicatie, die in taal, materiaal en aanpak aansluit bij de voorkeur en vaardigheden van de doelgroep.

#### 2 Werk samen met co-onderzoekers en/of ervaringsdeskundigen

- Neem ervaringsdeskundigen serieus. Maak ze écht onderdeel van het team én van je organisatie. Dat betekent onder andere dat jullie samen vergaderen en deelnemen aan teamuitjes, dat je oog hebt voor hun persoonlijke ontwikkeling en ze een passende vergoeding biedt, inclusief een kerstpakket.
- Overweeg de rollen waarin en manieren waarop je ervaringsdeskundigen bij het onderzoek betrekt en bespreek deze samen. Door meerdere rollen te realiseren – die van co-onderzoeker, meedenker bij het duiden van resultaten of communicatieadviseur – kun je meerdere mensen op verschillende manieren bij het onderzoek betrekken, wat leidt tot diversiteit en een grotere representativiteit.
- Samenwerken met ervaringsdeskundigen gaat vanuit een respectvolle en gelijkwaardige relatie. Weet dat het tijd kost om onderling (zelf)vertrouwen op te bouwen en houd daar in je planning en budget rekening mee [6, 7, 9, 10].
- Besteed in het team aandacht aan de competenties van zowel de onderzoeker(s) als de ervaringsdeskundigen die nodig zijn om mee samen te werken. Deze zijn reeds in eerder onderzoek in kaart gebracht en we benadrukken hier voor ons project de belangrijkste [6, 9, 10]: 1) een goede onderlinge communicatie, waarin een open en veilige sfeer, oog voor ieders behoeften en vaardigheden, en duidelijke afspraken over de aanpak en inhoud van de samenwerking centraal staan, 2) verwachtings-management tijdens verschillende fasen van het onderzoek en 3) reflectie op de samenwerking in praktische en emotionele zin.
- Digitaal samenwerken kan met mensen met LVB en/of laaggeletterden, mits dit zo makkelijk mogelijk wordt gemaakt door technologische en praktische ondersteuning.

#### 3 Werk samen met praktijkprofessionals

- Benut de expertise en praktijkkennis van professionals om de resultaten te duiden en te verrijken. Door deze verbinding tussen wetenschap en praktijk sluit de wetenschappelijke opbrengst aan bij de dagelijkse praktijk. De onderwerpen komen mede voort uit praktijkbehoeften en handelingsperspectieven kunnen gezamenlijk geformuleerd worden.
- Professionals en hun netwerk vormen een essentiële ingang bij het werven en bereiken van de doelgroep van mensen met LVB en/of laagge-letterden. Investeer daarom in dit netwerk, onder-houd contact tijdens de voortgang van het project en houd rekening met de impact op de werkvloer. Maak duidelijk waarom het voor deze medewerkers belangrijk is om tijd te investeren. Dat vraagt weer andere communicatiemiddelen dan richting de doelgroep.
- Reserveer voor het bij het onderzoek betrekken van praktijkprofessionals voldoende tijd en middelen in je project(begroting).

#### 4 Deel je voortgang en resultaten op een passende manier

- Deel je bevindingen zo veel mogelijk al gedurende het project en maak daarbij gebruik van verschillende kanalen en manieren voor wetenschappers, praktijkprofessionals en de doelgroep. Terugkoppeling over het onderzoek is belangrijk om mensen bij de uitvoering betrokken te houden en hen te laten zien wat er met hun input gebeurt.
- Zorg bij de terugkoppeling voor herkenbaarheid van je project en zorgvuldigheid in taalgebruik, zodat de doelgroep zich aangesproken voelt. In ons project gebruikten wij bijvoorbeeld ‘mensen die moeite hebben met lezen en schrijven’ of ‘mensen met LVB *en/of* laaggeletterden’ in onze communicatie, omdat niet iedereen zich door dergelijke termen aangesproken voelt, en mensen met LVB niet per se laaggeletterd zijn en andersom.
- Deel regelmatig in welke fase het project zich bevindt en wat van de deelnemers of andere betrokkenen verwacht wordt. Zo hebben we alle deelnemers een speciale bedankkaart gestuurd om te laten weten dat het onderzoek was afgerond.

## Conclusie

In reguliere onderzoeken zijn mensen die moeite hebben met de Nederlandse taal, zoals mensen met LVB en/of laaggeletterden, niet of nauwelijks vertegenwoordigd. Hierdoor ontbreekt structureel een belangrijke onderzoekspopulatie, wat resulteert in een onvolledig beeld van de volksgezondheid. De disproportionele effecten van de COVID-pandemie op de mentale gezondheid van mensen met LVB en/of laaggeletterden die ons onderzoek inzichtelijk maakte, onderstrepen de relevantie van inclusief volksgezondheidsonderzoek. Alleen door hun perspectief mee te nemen kan ook het zorgbeleid inclusief beleid worden.

Dit project laat zien dat het mogelijk is om deze doelgroep bij een gezondheidsmonitor te betrekken. Degenen die tot deze groep behoren, vinden het belangrijk dat hun stem gehoord wordt. Bovendien wijzen onze bevindingen erop dat een toegankelijke monitor ook voor een brede onderzoekspopulatie ingezet kan worden. Dankzij de succesfactoren in dit project weten we nog beter hoe we dit kunnen realiseren: betrek het perspectief van de beoogde doelgroep en Toegankelijk monitoronderzoek voor mensen met een lichte verstandelijke beperking en/of laaggeletterden stakeholders bij het opzetten en uitvoeren van onder-zoek, zodat dit zo veel mogelijk bij hun vaardigheden en leefwereld aansluit.

## References

[1] Jahagirdar D, Kroll T, Ritchie K (2013). Patient-reported outcome measures forchronicobstructive pulmonary disease: the exclusion of people with low literacy skills and learning disabilities. Patient.

[2] Krahn GL (2019). A call for better data on prevalence and health surveillance of people with intellectual and developmental disabilities. Intellect Dev Disabil.

[3] Rijksinstituut voor Volksgezondheid en Milieu (2022). Welbevinden en leefstijl tijdens de coronacrisis.

[4] Raaijmakers F (2020). De impact van COVID-19 onder laaggeletterden en mensen met een LVB: maatregelen, (mentale) gezondheid en handelingsperspectieven ten aanzien van de zorgen ondersteuningsbehoefte en beleid. ZonMw-project.

[5] Walmsley J, Johnson K (2003). Inclusive research with people with learning disabilities: past, present and futures.

[6] Frankena TK, Naaldenberg J, Cardol M (2019). A consensus statement on how to conduct inclusive health research. J Intellect Disabil Res.

[7] Wit de M, Bloemkolk D, Teunissen T (2016). Voorwaarden voor succesvolle betrokkenheid van patiënten/cliënten bij medisch wetenschappelijk onderzoek. TSG Tijdschr Gezondheidswet.

[8] Kooijmans R, Mercera G, Langdon P (2022). The adaptation of self-report instruments to the needs of people with intellectual disabilities: a systematic review. Clin Psychol.

[9] Embregts PJCM, Taminiau EF, Heerkens L (2018). Collaboration in inclusive research: competencies considered important for people with and without intellectual disabilities. J Policy Pract Intellect Disabil.

[10] Vlot-van Anrooij K, Frankena TK, Cruijsen van der A (2022). Shared decision making in inclusive research: reflections from an inclusive research team. Br J Learn Disabil.

